# Topical Chlorhexidine 0.2% versus Topical Natamycin 5% for the Treatment of Fungal Keratitis in Nepal: A Randomized Controlled Noninferiority Trial

**DOI:** 10.1016/j.ophtha.2021.12.004

**Published:** 2022-05

**Authors:** Jeremy J. Hoffman, Reena Yadav, Sandip D. Sanyam, Pankaj Chaudhary, Abhishek Roshan, Sanjay K. Singh, Sanjay K. Singh, Sailesh K. Mishra, Simon Arunga, Victor H. Hu, David Macleod, Astrid Leck, Matthew J. Burton

**Affiliations:** 1 International Centre for Eye Health, London School of Hygiene and Tropical Medicine, London, United Kingdom; 2 Sagarmatha Choudhary Eye Hospital, Lahan, Nepal; 3 Kilimanjaro Christian Medical Centre, Moshi, Tanzania; 4 Eastern Region Eye Care Programme, Biratnagar, Nepal; 5 Nepal Netra Jyoti Sangh, Kathmandu, Nepal; 6 Mbarara University of Science and Technology, Mbarara, Uganda; 7 International Statistics & Epidemiology Group, London School of Hygiene & Tropical Medicine, London, United Kingdom; 8 National Institute for Health Research Biomedical Research Centre for Ophthalmology at Moorfields Eye Hospital NHS Foundation Trust and UCL Institute of Ophthalmology, London, United Kingdom

**Keywords:** Chlorhexidine, Clinical trial, Corneal ulcer, Fungal keratitis, Natamycin, Nepal, BSCVA, best spectacle-corrected visual acuity, CI, confidence interval, DSMB, Data Safety and Monitoring Board, ED, epithelial defect, FK, fungal keratitis, logMAR, logarithm of the minimum angle of resolution, MK, microbial keratitis, MUTT, Mycotic Ulcer Treatment Trials, NPL, no perception of light, OR, odds ratio, SCEH, Sagarmatha Choudhary Eye Hospital, TPK, therapeutic penetrating keratoplasty, w/v, weight/volume

## Abstract

**Purpose:**

To investigate if topical chlorhexidine 0.2%, which is low cost and easy to formulate, is noninferior to topical natamycin 5% for the treatment of filamentous fungal keratitis.

**Design:**

Randomized controlled, single-masked, noninferiority clinical trial.

**Participants:**

Adults attending a tertiary-level ophthalmic hospital in Nepal with filamentous fungal infection confirmed on smear or confocal microscopy.

**Methods:**

Participants were randomly allocated to receive topical chlorhexidine 0.2% or topical natamycin 5%. Primary analysis (intention-to-treat) was by linear regression, using baseline logarithm of the minimum angle of resolution (logMAR) best spectacle-corrected visual acuity (BSCVA) and treatment arm as prespecified covariates. Mixed fungal-bacterial infections were excluded from the primary analysis but included in secondary analyses and secondary safety-related outcomes. The noninferiority margin was 0.15 logMAR. This trial was registered with ISRCTN, number ISRCTN14332621.

**Main Outcome Measures:**

The primary outcome measure was BSCVA at 3 months. Secondary outcome measures included perforation or therapeutic penetrating keratoplasty by 90 days.

**Results:**

Between June 3, 2019, and November 9, 2020, 354 eligible participants were enrolled and randomly assigned: 178 to chlorhexidine and 176 to natamycin. Primary outcome data were available for 153 and 151 of the chlorhexidine and natamycin groups, respectively. Of these, mixed bacterial-fungal infections were found in 20 cases (12/153 chlorhexidine, 8/151 natamycin) and excluded from the primary analysis. Therefore, 284 patients were assessed for the primary outcome (141 chlorhexidine, 143 natamycin). We did not find evidence to suggest chlorhexidine was noninferior to natamycin and in fact found strong evidence to suggest that natamycin-treated participants had significantly better 3-month BSCVA than chlorhexidine-treated participants, after adjusting for baseline BSCVA (regression coefficient, −0.30; 95% confidence interval [CI], −0.42 to −0.18; *P* < 0.001). There were more perforations and emergency corneal grafts in the chlorhexidine arm (24/175, 13.7%) than in the natamycin arm (10/173, 5.8%; *P =* 0.018, mixed infections included), whereas natamycin-treated cases were less likely to perforate or require an emergency corneal graft, after adjusting for baseline ulcer depth (odds ratio, 0.34; 95% CI, 0.15–0.79; *P =* 0.013).

**Conclusions:**

Treatment with natamycin is associated with significantly better visual acuity, with fewer adverse events, compared with treatment with chlorhexidine. Natamycin remains the preferred first-line monotherapy treatment for filamentous fungal keratitis.

Microbial keratitis (MK) is a severe, frequently blinding corneal infection. In tropical regions, fungal keratitis (FK) accounts for more than half of MK, with an estimated global annual incidence of approximately 1 million cases.^1^^,^^2^ The incidence is higher in low- and middle-income countries,^1^ particularly among agricultural workers.^3^ In temperate regions, although less common than bacterial keratitis, FK is increasing, with infection associated with contact lens use.^1^, ^2^, ^3^

Fungal keratitis is challenging to manage; there are significant barriers for patients accessing treatment, resulting in delays and poor outcomes.^4^^,^^5^ This is exacerbated by indiscriminate use of harmful topical corticosteroids and traditional eye medicines.^6^ The preferred treatment for FK is topical natamycin 5% based on the results of the Mycotic Ulcer Treatment Trials (MUTT), 3 additional clinical trials, and a systematic review favoring natamycin over voriconazole^7^, ^8^, ^9^, ^10^, ^11^; however, despite treatment with natamycin, some reports suggest approximately one quarter of patients continue to progress to corneal perforation and ultimately blindness or evisceration/enucleation.^4^^,^^7^^,^^12^ Despite recently being designated as an essential medicine by the World Health Organization,^13^ natamycin is not available in most countries in sub-Saharan Africa, as well as some countries in Asia and Europe.^4^ It is relatively expensive and difficult to formulate. Additional alternative and affordable medications are needed.

Chlorhexidine is a broad-spectrum, antiseptic biocidal agent that kills microorganisms through cell membrane disruption.^14^ It has been used in various forms in ophthalmology for >30 years, including as an eye drop preservative, for sterilizing contact lenses, and for treating fungal and *Acanthamoeba* keratitis.^15^^,^^16^ In the early 1990s, chlorhexidine 0.2% was reported effective in vitro against fungal isolates in an Indian case series.^17^ A systematic review of 2 subsequent small, underpowered randomized controlled trials found a nonsignificant trend favoring chlorhexidine over natamycin in “curing” by 21 days (relative risk, 0.70; 95% confidence interval [CI], 0.45–1.09).^11^^,^^18^^,^^19^ Chlorhexidine has the additional advantages of being inexpensive, easy to formulate, and generally well tolerated. In view of the clinical equipoise surrounding using natamycin or chlorhexidine for FK, we hypothesized that chlorhexidine might be noninferior to natamycin for the treatment of FK.

## Methods

### Study Design

We performed a randomized controlled, single-masked, noninferiority clinical trial comparing outcomes in patients with FK receiving natamycin 5% or chlorhexidine 0.2%. The trial was conducted at Sagarmatha Choudhary Eye Hospital (SCEH), Lahan, Nepal. The SCEH is a large, tertiary-level referral eye hospital, with 14 satellite eye care clinics that refer patients to SCEH. It serves a population of approximately 5 million people in Nepal. Additionally, because SCEH is 17 km from the Indian border, approximately half of patients are Indian nationals.

Ethical and regulatory approval was obtained from the Nepal Health Research Council Ethics Committee, the Nepal Department of Drug Administration, and the London School of Hygiene and Tropical Medicine Ethics Committee, United Kingdom. The study adhered to the principles of the Declaration of Helsinki. The trial protocol has been published.^20^ The trial was registered with ISRCTN, number ISRCTN14332621.

### Participants

Eligible patients were adults with acute MK (corneal epithelial ulceration >1 mm in diameter, corneal stromal infiltrate, and signs of acute inflammation) with evidence of filamentous fungal infection on in vivo confocal microscopy (by visualization of fungal hyphae) or smear microscopy (by visualization of fungal elements on potassium hydroxide, calcofluor white, or Gram stain). Exclusion criteria are listed in full in Table S1 (available at www.aaojournal.org) and included prior topical antifungal use, no perception of light visual acuity in the affected eye, and very severe ulcers warranting immediate surgical intervention (e.g., impending perforations and perforated corneal ulcers).

Recruitment was in 2 stages to facilitate data collection on all potential participants at baseline before FK diagnosis was confirmed. All consenting adult patients with signs of acute MK were eligible for Stage 1 before microscopy (in vivo confocal microscopy and smear after corneal scrape) to confirm the type of infection. In vivo confocal microscopy diagnosis of FK was performed by experienced operators. Corneal scrapes were also performed for microbiological cultures for fungal organism identification and to diagnose any mixed bacterial-fungal infections. Only consenting people with filamentous fungal infection meeting the criteria in Table S1 were eligible for Stage 2 and inclusion in the trial. These participants then underwent masked intervention assignment.

### Randomization and Masking

Participants were randomly assigned (1:1) to chlorhexidine 0.2% or natamycin 5%. A computer-generated randomization list was prepared by an independent statistician using Stata 16 (StataCorp LP) and random block sizes of 2, 4, or 6. Treatment allocation was concealed in sequentially numbered, opaque envelopes. The random allocation procedure was conducted by an independent randomization administrator with no other involvement in the trial.

Given that the topical treatments under investigation in this study have different appearances (chlorhexidine is a clear, colorless solution, whereas natamycin is an opaque, white suspension), it was not possible to mask participants; however, patients were not told which treatment they were allocated to, and bottle labels were replaced with standardized labels with “A” or “B” replacing the drug name. Clinicians were masked to treatment allocation, with a nurse not otherwise involved in the trial ensuring that any natamycin residue was removed before examination.^7^ Compliance was checked through self-reporting and weighing of study medication bottles. The statistician performing the primary analysis was masked to allocation and only received the allocation sequence after the analysis code was prepared and pretested. Best spectacle-corrected visual acuity (BSCVA) at 3 months (primary outcome measure) was assessed by an optometrist masked to treatment allocation and not otherwise involved in any other aspects of the trial.

### Procedures

Patients were randomized to chlorhexidine 0.2% weight/volume (w/v) (preservative-free, aqueous dilution including unspecified buffer, produced by Mandeville Medicines) or natamycin 5% w/v (preserved with benzalkonium chloride 0.01% and manufactured by FDC Pharmaceuticals Ltd). Dosing schedules were identical between arms and consisted of 1 drop applied to the affected eye every 1 hour per day and night for 48 hours, then hourly while awake for 5 days, then every 2 hours while awake until 3 weeks from enrollment. Further continuation and treatment duration of the masked medication depended on the clinical response. If the ulcer had healed (epithelial defect [ED] <1 mm, infiltrate resolved, with or without corneal scarring), then the treatment was stopped. If there was resolving stromal infiltration or ED >1 mm but <5 mm, treatment was reduced to 4 times daily. If the stromal infiltration or hypopyon was resolving, but the ED was still > 5 mm, treatment was reduced to 6 times daily. All antifungal medications were kept in a dark place at <25 ° C. Topical medications were replenished as needed at follow-up. For ethical reasons, ophthalmologists added or changed any adjunctive medications if deemed clinically necessary, including ocular antihypertensives for raised intraocular pressure and topical mydriatics for cycloplegia and pain relief. Ophthalmologists prescribed topical antibiotics (moxifloxacin 0.5% w/v) if there was evidence of a mixed bacterial-fungal infection (based on microbiological culture results reported to the ophthalmologist from baseline or subsequent corneal scrapes) or if the ED was >5 mm, as per local protocols.

In patients with progressive FK despite 7 days or more of trial medication, ancillary treatment was considered. After repeating microbiological tests to rule out a mixed bacterial-fungal infection, deteriorating patients with superficial infiltration were started on topical voriconazole 1% hourly in addition to their trial medication. For infiltrates involving >75% of corneal thickness on slit-lamp examination, oral ketoconazole 200 mg twice daily was added (with monitoring of liver function). Ancillary treatment choices were limited by local availability. If, after 7 days of additional treatment, despite these measures, there was progression, surgical management such as therapeutic penetrating keratoplasty (TPK) was contemplated. The decision to perform TPK or not rested with the ophthalmologist, who considered factors including location and depth of the ulcer, limbal involvement, and size of the perforation.

Participants were counseled before enrollment and advised to return to SCEH in the event of any concerns or worsening symptoms rather than attend alternative facilities. Patients were directly asked whether they had visited other health care facilities (including pharmacists and traditional healers) at each follow-up.

### Outcomes

Patients were assessed at baseline and at 2, 7, 14, 21, 60, and 90 days postenrollment. The BSCVA was measured at enrollment and at 90 days by a masked trial-certified optometrist. The BSCVA protocol followed was that used in the MUTT and the Steroids for Corneal Ulcers Trials studies,^7^^,^^12^ which was adapted from the Age-Related Eye Disease Study using a 3 m, proportionally reduced version of the 4 m Early Treatment Diabetic Retinopathy Study tumbling “E” chart (Good-Lite) at 3 m,^21^ with low-vision testing at 0.75 m. Presenting and pinhole visual acuity were also measured at each visit by trial-certified eye health workers using Peek Acuity, a validated smartphone application.^22^

The primary outcome measure was BSCVA at day 90. Secondary outcome measures included presenting (unaided) visual acuity at 21 and 90 days; scar/infiltrate size at 7, 21, and 90 days; time to full epithelial healing (defined as the midpoint between the preceding visit when an ED was present and the subsequent visit when the ED was absent or measured <0.5 mm); microbiological cure rate (baseline culture-positive patients who became culture-negative at day 7); ulcer depth at 7 and 21 days; hypopyon height at 7 and 21 days; perforation or TPK by 90 days; loss of eye by 90 days; and ocular adverse events.

A calibrated slit-lamp biomicroscope was used to assess infiltrate or scar size, ED, depth, hypopyon, and ocular adverse events at each follow-up. Measurements of infiltrate, scar, and ED involved measuring the longest dimension and longest perpendicular,^7^^,^^12^ a protocol previously adapted from the Herpetic Eye Disease Study.^23^ Reepithelialization was defined as the absence of an ED with the administration of fluorescein. Depth was assessed in 4 categories: >0% to 25%, >25% to 50%, >50% to 75%, and >75%. All grading ophthalmic clinicians were trial certified and masked to treatment allocation. Patients were monitored at each visit for adverse events or drug reactions. We followed standard definitions for adverse events, including drug toxicity.

### Statistical Analysis

Analyses followed a predetermined plan.^20^ The trial was powered to test the hypothesis that chlorhexidine is noninferior to natamycin with respect to the primary outcome at a prespecified noninferiority margin of 0.15 logarithm of the minimum angle of resolution (logMAR) units. A sample size of 500 patients (250 per arm) was fixed before enrollment and estimated to provide 90% power to detect a 0.15 logMAR difference in BSCVA at 3 months between arms, assuming ±0.5 standard deviation for 3 months BSCVA, a type I error rate of 5%, 10% mixed bacterial/fungal infections and 15% drop-out, and a single interim analysis.

Baseline characteristics were summarized by arm. Linear regression was used for primary analysis of the primary outcome, with treatment arm and baseline BSCVA as prespecified covariates, excluding mixed infections. Primary analysis was by intention-to-treat. Secondary analyses of the primary outcome included a per-protocol analysis and analysis of the primary outcome (by intention-to-treat) including mixed infections.

For secondary outcome analysis, the geometric mean of the longest diameter and the longest perpendicular was used to assess infiltrate or scar size and ED size.^7^ Analysis was by linear regression for infiltrate or scar size, using treatment arm and infiltrate or scar size at baseline as covariates. Linear regression was used for ulcer depth and hypopyon height, with treatment arm and baseline ulcer depth as covariates. Time to full epithelial healing was analyzed using a Cox proportional hazards model, with treatment arm and baseline ED size as covariates. Adverse events between arms were compared by Fisher exact test. A logistic regression model with covariates for treatment arm and baseline infiltrate depth was used to assess the odds of corneal perforation or TPK.

For patients who underwent TPK, we assigned a 3-month logMAR of 1.9.^7^ For infiltrate, scar, and ED size, we used the most recent value before surgery. Sensitivity analyses for patients lost to follow-up were conducted using linear mixed-effects regression, including all outcomes measured for each patient. All analyses were conducted using Stata 16 (StataCorp LLC).

A Data Safety and Monitoring Board (DSMB) was established before enrollment to oversee safety, data quality, and trial conduct. The DSMB met every 6 months during recruitment. One planned interim analysis was conducted after one third of all planned patients (167/500) had completed their 90-day follow-up and presented to the DSMB. After this initial interim analysis, recruitment was paused by the trial steering committee and endorsed by the DSMB, with advice to perform a second (unplanned) interim analysis that included all patients who had completed their 90-day follow-up to that date (319/500). After the second interim analysis, recruitment was stopped at 354 participants. The 35 patients under active management completed their allocated treatment and follow-up.

## Results

**Figure 1:**
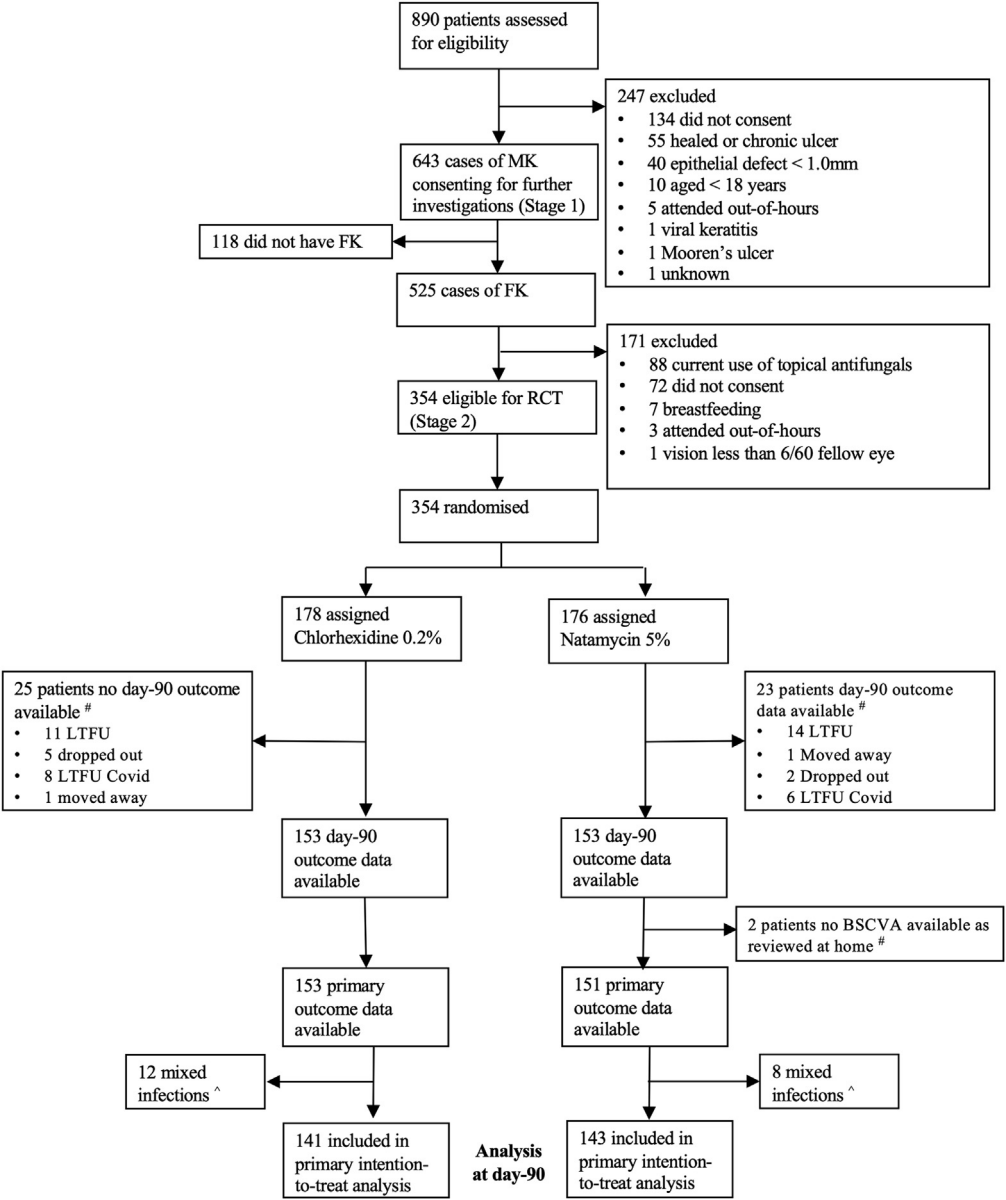
Trial profile. A total of 135 patients physically attended clinic for their 90-day follow-up in the chlorhexidine 0.2% arm, with additional visual acuity outcome data available in 18 patients (because they had undergone therapeutic penetrating keratoplasty or eye removal surgery), and 145 patients physically attended clinic for their 90-day follow-up in the natamycin 5% (NATA) arm, with additional visual acuity outcome data available in 8 patients (because they had undergone therapeutic penetrating keratoplasty or eye removal surgery or had no perception of light vision in the affected eye due to acute glaucoma). Ninety-day best spectacle-corrected visual acuity (BSCVA) outcome data were unavailable in 2 patients who attended in the NATA arm because these patients were reviewed at home. ˆ Mixed fungal-bacterial infections are excluded from the primary analysis but included in the secondary analysis. There were 25 mixed infections in total (15 in the chlorhexidine 0.2% arm, 10 in the NATA arm) at baseline. At the day 90 follow-up, outcome data were available for 12 mixed infections in the chlorhexidine 0.2% arm and for 8 in the NATA arm. Mixed infections included the following: filamentous fungus plus any of gram-positive cocci (n = 9), gram positive bacilli (n = 6), gram negative cocci (n = 2), *Staphylococcus aureus* (n = 3), *Streptococcus pneumoniae* (n = 2), *Corynebacterium spp.* (n = 1), and *Streptococcus spp.* (n = 2). COVID = coronavirus; FK = fungal keratitis; LTFU = lost to follow-up; MK = microbial keratitis; RCT = randomized controlled trial. # Ninety-day best spectacle-corrected visual acuity (BSCVA) outcome data were unavailable in 2 patients who attended in the NATA arm because these patients were reviewed at home.

Between June 3, 2019, and November 9, 2020, 890 patients with suspected MK were assessed. Of these, 643 were confirmed to have clinical features of acute MK and consented to undergo further investigations (Stage 1). Smear or confocal microscopy identified 525 cases of filamentous FK, of which 354 patients (67.2%) met eligibility criteria and consented for enrollment in the clinical trial (Stage 2). Reasons for exclusion are given in Figure 1. All 354 eligible patients were randomized; 178 were allocated to chlorhexidine, and 176 were allocated to natamycin. Recruitment and follow-up were paused on March 24, 2020, and resumed on June 13, 2020, because of regulatory restrictions relating to the coronavirus disease 2019 pandemic. There were 46 patients who completed the study whose final review was originally scheduled during this period; 33 of 46 patients were delayed by >30 days because of the restrictions; however, there was no evidence of a difference between arms (*P* = 0.3311). Recruitment was stopped on November 9, 2020, after the interim analysis and guidance from the DSMB. Follow-up was completed on February 8, 2021. There were 25 and 23 patients for whom day 90 outcome data were not available in the chlorhexidine and natamycin arms, respectively. An additional 2 patients were seen at home in the natamycin group; therefore, 90-day BSCVA was unavailable. Mixed bacterial-fungal infections were present in 15 of 178 patients (8.4%) and 10 of 176 patients (5.7%) at baseline, with 90-day outcome data available for 12 of 178 patients and 8 of 176 patients randomized to chlorhexidine or natamycin, respectively. Therefore, we included 141 patients randomized to chlorhexidine and 143 patients randomized to natamycin in the primary analysis (Fig 1).

The mean age of enrolled participants was 46.7 years (standard deviation ± 13.3), and 219 of 354 (61.9%) were female. Baseline demographic and clinical characteristics were generally well balanced between groups (Table 1). Most clinical features, including infiltrate size, ulcer depth, and presence of hypopyon, were similar across the 2 groups; however, in terms of visual acuity, there were more “blind” eyes in the chlorhexidine group than in the natamycin group (40 vs. 27 with visual acuity worse than 3/60).

**Table 1:** Baseline Demographic and Clinical Characteristics for All Enrolled Patients (including Mixed Infections)

	Chlorhexidine (n = 178)		Natamycin (n = 176)		Total (N = 354)	
**DemographicFeatures**						
Age, yrs	46.1	(13.5)	48.2	(13.0)	46.9	(13.3)
Sex						
Male	73	(41.0%)	62	(35.2%)	135	(38.1%)
Female	105	(59.0%)	114	(64.8%)	219	(61.9%)
Literacy						
Illiterate	139	(78.1%)	138	(78.4%)	277	(78.3%)
Little Nepali (read/write)	16	(8.99%)	14	(7.95%)	30	(8.47%)
Nepali well (read/write)	9	(5.06%)	15	(8.52%)	24	(6.78%)
English and Nepali (read/write)	14	(7.87%)	9	(5.11%)	23	(6.50%)
Marital status						
Single	6	(3.37%)	3	(1.70%)	9	(2.54%)
Married	161	(90.5%)	164	(93.2%)	325	(91.8%)
Divorced	2	(1.12%)	3	(1.70%)	5	(1.41%)
Widowed	9	(5.06%)	6	(3.41%)	15	(4.24%)
Occupation						
Agriculture	91	(51.1%)	98	(55.7%)	189	(53.4%)
Nonagriculture∗	87	(48.9%)	78	(44.3%)	165	(46.6%)
Trauma						
Vegetative matter/wood	73	(41.0%)	73	(41.5%)	146	(41.2%)
Other†	19	(10.7%)	18	(10.2%)	37	(10.5%)
Unknown object	2	(1.12%)	1	(0.06%)	3	(0.85%)
Contact lens	0	(0%)	0		0	
No history of trauma/unknown	84	(47.2%)	84	(47.7%)	168	(47.5%)
Previous treatment						
No	32	(18.0%)	28	(15.9%)	60	(17.0%)
Yes‡	146	(82.0%)	148	(84.1%)	294	(83.1%)
Previous topical steroids	33	(18.5%)	26	(14.8%)	59	(16.7%)
Previous TEM	2	(1.12%)	3	(1.7%)	5	(1.41%)
Previous antibiotics	120	(67.4%)	129	(73.3%)	249	(70.3%)
Previous other topical§	70	(39.3%)	60	(34.1%)	130	(36.7%)
Previous systemic medication	88	(49.4%)	81	(46.0%)	169	(47.7%)
**Clinical Features**						
Laterality						
Right	78	(43.8%)	89	(50.6%)	167	(47.2%)
Left	100	(56.2%)	87	(49.4%)	187	(52.8%)
BSCVA						
Mean (logMAR)	0.65	(0.62)	0.56	(0.57)	0.61	(0.60)
Median (logMAR) ˆ	0.45	(0.12–1.00)	0.38	(0.12–0.80)	0.40	(0.12–0.90)
6/5–6/12	61	(34.27%)	76	(43.18%)	137	(38.70%)
>6/12–6/18	40	(22.47%)	31	(17.61%)	71	(20.06%)
>6/18–6/60	34	(19.10%)	39	(22.16%)	73	(20.62%)
>6/60–3/60	3	(1.69%)	3	(1.70%)	6	(1.69%)
>3/60–1/60 (CF)	36	(20.22%)	22	(12.50%)	58	(16.38%)
>1/60 (CF) no light perception	4	(2.25%)	5	(2.84%)	9	(2.54%)
Contrast sensitivity ˆ	0.98	(0.45–1.20)	1.05	(0.75– 1.35)	1.05	(0.60–1.35)
Baseline infiltrate size (mm)						
Median ˆ	2.55	(1.75–3.70)	2.50	(1.68–3.70)	2.50	(1.75–3.70)
Median ˆ	2.55	(1.75–3.70)	2.50	(1.68–3.70)	2.50	(1.75–3.70)
≤0.5	0	(0%)	1	(0.57%)	1	(0.28%)
>0.5–1.5	30	(16.85%)	32	(18.18%)	62	(17.51%)
>1.5–2.5	58	(32.58%)	59	(33.52%)	117	(33.05%)
>2.5–3.5	36	(20.22%)	35	(19.89%)	71	(20.06%)
>3.5–4.5	26	(14.61%)	26	(14.77%)	52	(14.69%)
>4.5–5.5	13	(7.30%)	11	(6.25%)	24	(6.78%)
>5.5–6.5	5	(2.81%)	5	(2.84%)	10	(2.82%)
>6.5–7.5	5	(2.81%)	4	(2.27%)	9	(2.54%)
>7.5–8.5	1	(0.56%)	1	(0.57%)	2	(0.56%)
>8.5–9.5	2	(1.12%)	2	(1.14%)	4	(1.13%)
>9.5	2	(1.12%)	0	(0%)	2	(0.56%)
ED, mm ˆ	2.75	(2.05–3.90)	2.60	(1.90–3.78)	2.70	(2.00–3.80)
Ulcer depth						
1%–25%	117	(65.7%)	114	(64.8 %)	231	(65.3%)
26%–50%	54	(30.3%)	56	(31.8%)	110	(31.1%)
51%–75%	5	(2.81%)	6	(3.41%)	11	(3.11%)
76%–100%	2	(1.12%)	0		2	(0.56%)
Presence of hypopyon	37	(20.8%)	32	(18.2%)	69	(19.5%)
Hypopyon height, mm ˆ ‖	0.5	(0.3–1.0)	0.6	(0.2–1.0)	0.5	(0.3–1.0)
Time from symptoms to presentation, days	8.13	(5.93)	8.36	(8.35)	8.25	(7.22)
Time from trauma to presentation, days	8.68	(6.14)	9.00	(8.09)	8.84	(7.16)
Ocular surface disease¶	3	(1.69%)	6	(3.41%)	9	(2.54%)
Dacryostenosis or dacryocystitis#	7/168	(4.17%)	3/168	(1.79%)	10/336	(2.98%)
Preexisting corneal abnormalities	0		0		0	
Preexisting eyelid or eyelash abnormalities∗∗	3	(1.69%)	2	(1.14%)	5	(1.41%)
Diabetes mellitus††	1	(0.56%)	4	(2.27%)	5	(1.41%)

Data are n (%) or mean (standard deviation), other than where indicated with "ˆ" when the data are median (interquartile range).

BSCVA = best spectacle-corrected visual acuity; CF = counting fingers; ED = epithelial defect; logMAR = logarithm of the minimum angle of resolution; TEM = traditional eye medicine.

∗ Includes unemployed, retired, and so forth.

† Includes soil, dust, insect, cow’s tail, fingernail, chemicals, and clothes.

‡ Some patients were receiving >1 medication at enrollment.

§ Includes dilating eyedrops, lubricating eyedrops, topical antivirals, topical nonsteroidal anti-inflammatory drugs, and glaucoma medication.

‖ If present.

¶ Represents patients who had moderate to severe dry eye with significant punctate epithelial erosions, conjunctival scarring resulting from cicatrizing conjunctivitis or chemical burns, allergic eye disease, and so forth. It does not include patients with blepharitis alone.

# No enrolled patients had a history of dacrocystitis or had undergone a surgical procedure for dacryostenosis before enrollment. Patients were offered nasolacrimal duct syringing as part of their clinical examination. The numbers therefore represent patients who were incidentally found to have dacryostenosis during their baseline clinical examination. Some patients refused to undertake lacrimal syringing, or it was not possible due to coronavirus disease 2019 policy (10 in chlorhexidine arm and 8 in natamycin arm).

∗∗ Includes entropion, lagophthalmos, and trichiasis.

†† Diabetes mellitus and human immunodeficiency virus infection were the only systemic diseases that were self-reported or investigated; there were no cases of human immunodeficiency reported or detected in study participants.

Fungal keratitis was diagnosed by in vivo confocal microscopy alone for 30 cases (8.5%), whereas microscopy only was positive in 27 cases (7.6%). The most commonly isolated organisms were *Curvularia* species (118/354, 33.3%), *Fusarium* species (47/354, 13.3%), and *Aspergillus* species (32/354, 9.0%). There was no growth in 47 of 354 (13.3%) microscopy positive cases, whereas there were 34 of 354 (9.6%) unidentified filamentous fungi due to nonsporulation in vitro or loss to contamination (Table S2 available at www.aaojournal.org).

**Figure 2:**
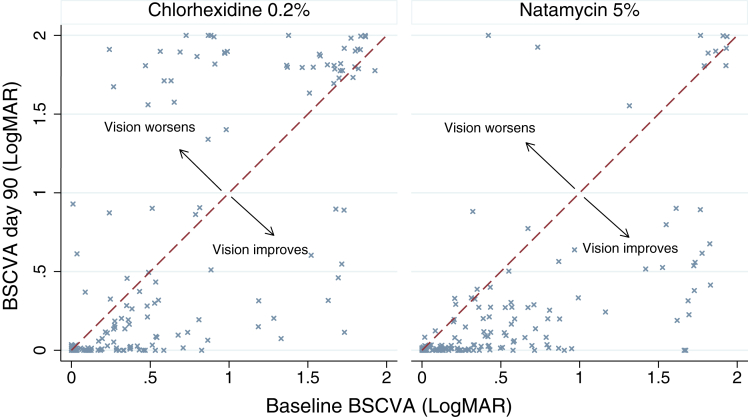
Ninety-day BSCVA versus baseline BSCVA for patients in each investigational arm (excluding mixed infections). Graph plotted with jitter added to prevent overlapping points. The red dashed line is where BSCVA at baseline and BSCVA at day 90 are the same. Note patients who have undergone a corneal graft (therapeutic penetrating keratoplasty) are allocated a BSCVA of 1.9 logarithm of the minimum angle resolution (logMAR) and those who have had their eye removed are allocated a BSCVA of 2.0 logMAR. BSCVA = best spectacle-corrected visual acuity; logMAR = logarithm of the minimum angle of resolution.

Among the 284 participants included in the primary analysis, the baseline mean BSCVA was 0.61 logMAR (95% CI, 0.51–0.70) in the chlorhexidine group and 0.55 logMAR (95% CI, 0.46–0.64) in the natamycin group. By day 90, this had changed to 0.64 logMAR (95% CI, 0.51–0.77) in the chlorhexidine arm and 0.26 logMAR (95% CI, 0.18–0.35) in the natamycin arm, which was a change of 0.03 logMAR and −0.29 logMAR, respectively. After adjusting for baseline BSCVA, we estimate that BSCVA among patients treated with chlorhexidine is approximately 3 lines worse than those treated using natamycin (regression coefficient, −0.30; 95% CI, −0.42 to −0.18). This provides no evidence (*P* = 1.00) that chlorhexidine is noninferior to natamycin and provides strong evidence (*P* < 0.001) that natamycin is superior to chlorhexidine (Fig 2). Visual acuity measurements were unavailable for 50 participants at 90 days; however, there was no evidence to suggest that loss to follow-up was associated with baseline visual acuity, baseline infiltrate size, age, gender, or treatment assignment (Table S3, available at www.aaojournal.org). Furthermore, if we used the last observation carried forward for 90-day BSCVA, the results were similar to the primary analysis (regression coefficient. −0.32; 95% CI, −0.21 to –0.43; *P* < 0.001). No participants who completed the study reported visiting other health care facilities (including traditional healers) or using additional medication after enrollment.

At 21 days, correcting for baseline BSCVA, there was no evidence of a difference in mean BSCVA between those allocated to chlorhexidine and those allocated to natamycin (regression coefficient, −0.088 logMAR; 95% CI, −0.18 to 0.059; *P* = 0.066). The 3-week mean BSCVA was 0.36 logMAR (95% CI, 0.28–0.44) in the natamycin arm compared with 0.50 logMAR (95% CI, 0.40–0.60) in the chlorhexidine arm; however, there was evidence of a difference in infiltrate or scar size between the 2 treatment arms at each follow-up interval. At day 7, the estimated mean infiltrate size was 0.26 mm (95% CI, −0.49 to −0.04; *P =* 0.022) smaller in the natamycin arm than in the chlorhexidine arm, adjusting for baseline infiltrate size. At 21 days, estimated mean infiltrate size was 0.42 mm (95% CI, −0.73 to −0.10; *P =* 0.009) smaller in the natamycin arm than in the chlorhexidine arm, adjusting for baseline infiltrate size. By 90 days, there remained evidence of a difference in scar/infiltrate size between patients randomized to the 2 treatments (regression coefficient, −0.40 mm; 95% CI, −0.57 to −0.23; *P* < 0.001), adjusting for baseline infiltrate size.

**Figure 3:**
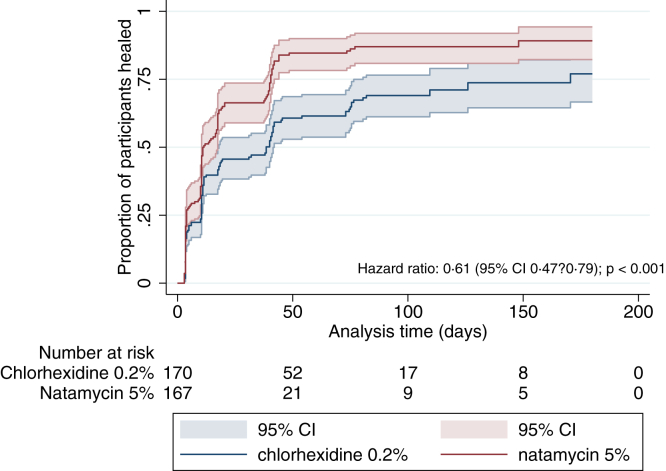
Kaplan–Meier survival curve plotting time to full epithelialization. Patients who had undergone a therapeutic penetrating keratoplasty or those who were eviscerated are included in this figure but are by definition “not healed.” The time goes beyond the day 90 final follow-up because some patients were reviewed beyond this time because of delays resulting from the coronavirus disease 2019 pandemic. Data were missing for 8 patients in the chlorhexidine 0.2% arm and 9 patients in the natamycin 5% arm. CI = confidence interval.

We found evidence of a difference in time to reepithelialization by treatment arm after controlling for baseline ED size through Cox proportional hazards regression (*P <* 0.001). Patients treated with chlorhexidine healed 39% more slowly than those treated with natamycin (hazard ratio, 0.61; 95% CI, 0.47–0.79) (Fig 3 and Fig S4 [available at www.aaojournal.org]). With regard to treatment failure, as defined by a persistent ED at 90-day follow-up of >0.5 mm, there were no patients in the natamycin group who had not reepithelialized compared with 11 of 122 (9.0%, *P <* 0.001; excluding mixed infections) in the chlorhexidine group.

After excluding mixed infections, there was evidence of a difference in hypopyon height at 1 week but not at 3 weeks in the patients who presented with a hypopyon, after controlling for baseline hypopyon height between arms (1-week regression coefficient, 0.46 mm; 95% CI, 0.024–0.89, *P =* 0.039; n = 59; 3-week regression coefficient, 0.19 mm; 95% CI, −0.21 to 0.59; *P =* 0.340; n = 56). Likewise, there was no evidence of a difference in ulcer depth at 1 or 3 weeks between arms after controlling for baseline depth (1-week regression coefficient, −0.92% of corneal thickness; 95% CI, −3.58 to 1.73 *P =* 0.495; 3-week regression coefficient, −1.42% of corneal thickness; 95% CI, −4.80 to 1.93; *P =* 0.405).

A slightly higher proportion of patients randomized to chlorhexidine remained culture positive at day 7 (22/83, 26.5%; 95% CI, 0.09–28.2) compared with those randomized to natamycin (11/65, 16.9%; 95% CI, 17.4–37.3), although this was not statistically significant (*P =* 0.232). This remained the case after adjusting for the baseline causative organism by logistic regression (odds ratio [OR], 2.21; 95% CI, 0.84–5.81; *P =* 0.107). The cultured fungal organisms were different at baseline and day 7 in 8 patients (Table S4, available at www.aaojournal.org). Similar results were obtained if negative results were assumed in the 208 patients for whom repeat culture was not performed (data not shown).

Clinical outcome and adverse events are summarized in Table 2 for all patients who attended at least 1 follow-up appointment (348/354), including those with mixed infections at baseline. A perforation developed or a TPK was required in 24 of 175 patients (13.7%) in the chlorhexidine arm compared with 10 of 173 patients (5.8%) in the natamycin arm (OR, 0.34; 95% CI, 0.15–0.79; *P =* 0.013, adjusting for baseline depth).

**Table 2:** Clinical Outcomes and Adverse Events by Treatment Group (including Mixed Infections)

	Chlorhexidine		Natamycin		Total		*P* Value
**Clinical Outcomes**							
Day 90 BSCVA (logMAR)							
Mean	0.64	(0.79)	0.26	(0.52)	0.45	(0.69)	<0.001∗
Median ˆ	0.2	(0–1.7)	0.02	(0–0.26)	0.1	(0–0.58)	NA
6/5–6/12	86/178	(48.31%)	119/176	(67.61%)	205/354	(57.91%)	<0.001†
>6/12–6/18	9/178	(5.06%)	8/176	(4.55%)	17/354	(4.80%)	
>6/18–6/60	10/178	(5.62%)	13/176	(7.39%)	23/354	(6.50%)	
>6/60–3/60	0		0		0		
>3/60–1/60 (CF)	26/178	(14.61%)	3/176	(1.70%)	29/354	(8.19%)	
>1/60 (CF)–no light perception	47/178	(26.40%)	33/176	(18.75%)	80/354	(22.60%)	
Day 90 visual acuity (presenting, BSCVA) ˆ	0.2	(0–1.5)	0	(0–0.3)	0.1	(0–0.4)	
Day 90 scar size (mm)							
Median ˆ	2.3	(1.75–3.3)	2.25	(1.5–3.35)	2.25	(1.6–3.3)	0.837†
≤2	49/118	(41.5%)	62/145	(42.8%)	111/263	(42.2%)	
>2–4	50/118	(42.4%)	65/145	(44.8%)	115/263	(43.7%)	
>4–6	15/118	(12.7%)	15/145	(10.3%)	30/263	(11.4%)	
>6	4/118	(3.4%)	3/145	(2.1%)	7/263	(2.7%)	
Day 7 hypopyon	19/162	(11.7%)	31/158	(19.6%)	50/320	(15.6%)	0.064†
Day 7 hypopyon height, mm (median) ˆ‡	0.8	(0.2–1)	0.5	(0.2–1.5)	0.55	(0.2–1.5)	NA
Day 7 hypopyon height (mean)‡	0.81	(0.62)	0.93	(0.99)	0.88	(0.86)	0.636§
Day 21 hypopyon	11/144	(7.6%)	18/149	(12.1%)	29/293	(9.9%)	0.242†
Day 21 hypopyon height (median) ˆ‡	1.4	(0.3–3)	1.1	(0.5–1.5)	1.2	(0.5–1.8)	NA
Day 21 hypopyon height (mean)‡	1.52	(1.25)	1.14	(0.82)	1.28	(1.00)	0.3299§
Day 7 culture positive	22/83	(26.5%)	11/65	(16.9%)	33/148	(22.3%)	0.232†

**Adverse Events**							
*Adverse Events–Serious*							
Corneal perforation	13/175	(7.47%)	6/173	(3.46%)	19/348	(5.45%)	0.101†
TPK	11/175	(6.28%)	4/173	(2.31%)	15/348	(4.31%)	0.111†
Corneal perforation or TPK	24/175	(13.7%)	10/173	(5.79%)	34/348	(9.77%)	0.018∗†
Evisceration‖	8/175	(4.6%)	3/173	(1.73%)	11/348	(3.2%)	0.219†
Endophthalmitis	0		0		0		NA
*Adverse Events–Nonserious*							
Local allergic reaction							
None	155/175	(88.6%)	165/173	(95.4%)	320/348	(92.0%)	0.048∗†
Mild	18/175	(10.3%)	8/173	(4.6%)	26/348	(7.47%)	
Moderate	1/175	(0.57%)	0/173		1/348	(0.29%)	
Severe	1/175	(0.57%)	0/173		1/348	(0.29%)	
>2-mm increase in hypopyon	3/175	(1.71%)	1/173	(0.57%)	4/348	(1.15%)	0.623†
>50% increase in infiltrate size¶	13/175	(7.42%)	3/173	(1.73%)	16/348	(4.60%)	0.019∗†
Progressive corneal thinning to ≤50%¶	13/175	(7.42%)	5/173	(2.89%)	18/348	(5.17%)	0.088†
New cataract development	13/175	(7.42%)	9/173	(5.20%)	22/348	(6.32%)	0.510†
Persistent ED	15/175	(8.57%)	0/173		15/348	(4.31%)	<0.00∗†
Corneal edema	30/175	(17.1%)	11/173	(6.36%)	41/348	(11.8%)	0.002∗†
Secondary bacterial keratitis during study¶#	49/175	(28.0%)	44/173	(25.4%)	93/348	(26.7%)	0.629†

Data are n (%) or mean (standard deviation), other than where indicated with "ˆ" when the data are median (interquartile range). There were no systemic side effects reported in either arm, including death, need for nonelective surgery or hospitalization, or myocardial infarction or stroke, and are therefore not presented here. There were no cases of intraocular pressure ≥35 mmHg for 1 week despite therapy in either arm.

BSCVA = best spectacle-corrected visual acuity; CF = counting fingers; ED = epithelial defect; logMAR = logarithm of the minimum angle of resolution; NA = not available; TPK = therapeutic penetrating keratoplasty.

The denominator represents patients who attended for at least 1 follow-up during the study period or who attended the follow-up review in question; patients who did not attend after enrollment or who did not attend the specified follow-up review (e.g., day 21) are treated as missing data and excluded from this analysis.

∗ *P* value calculated by linear regression after adjusting for baseline visual acuity.

† Calculated by Fisher exact test.

‡ If present.

§ Calculated by *t* test.

‖ All patients who were eviscerated had already perforated.

¶ From baseline.

# Secondary bacterial keratitis defined as a patient commencing a topical antibiotic during the study period because of clinical deterioration and clinical impression or as a patient who has microscopy from a corneal scrape during the study period with evidence of a bacterial infection.

By including mixed infections in analysis of the primary outcome, we estimate that the mean BSCVA among patients randomized to chlorhexidine was approximately 3.2 lines worse than those randomized to natamycin (regression coefficient, −0.32; 95% CI, −0.43 to −0.20; *P <* 0.001) after correcting for baseline BSCVA. Given that there were more patients who had received topical steroids in the chlorhexidine arm than in the natamycin arm, we performed a sensitivity analysis excluding these patients, although these results were similar to the primary analysis results (regression coefficient, −0.283; 95% CI, −0.47 to −0.16; *P <* 0.001).

The odds of poor BSCVA (defined as worse than 1.0 logMAR at day 90 follow-up) was estimated to be 10 times higher in the chlorhexidine group than in the natamycin group (OR, 10.2; 95% CI, 3.6–28.5; *P <* 0.001) after adjusting for baseline BSCVA (OR, 6.2; 95% CI, 2.8–13.7; *P <* 0.001) and baseline mean infiltrate size (OR, 1.73; 95% CI, 1.3–2.3; *P <* 0.001). There was an extremely strong association between baseline BSCVA and a poor outcome, with almost all those who had good vision at baseline having a good outcome. In fact, none of the participants in the quartile with the best baseline BSCVA or the quartile with the smallest baseline infiltrate size had a poor visual outcome regardless of treatment allocation (Table S5, available at www.aaojournal.org). This made the overall OR estimation imprecise, which can be seen from the wide CIs. This could suggest that both chlorhexidine and natamycin are effective for mild disease, as evidenced by a good outcome for all participants within the quartile of patients with the best vision at baseline and 95% of patients within the quartile with the smallest infiltrate size at baseline (Table S5, available at www.aaojournal.org), the difference being found mostly for patients presenting with severe disease. Alternatively, mild disease may be self-limiting and heal regardless of treatment given. Other potential determinants of success (including age, sex, presence of hypopyon, ED size at baseline, clinical history or demographic features, genus and species of fungus, or presence of mixed infection) were not significant and therefore excluded.

## Discussion

We tested the hypothesis that chlorhexidine 0.2% was noninferior to natamycin 5% for the treatment of FK; however, we found no evidence to support this. Visual acuity was significantly better at day 90 in participants randomized to natamycin than to chlorhexidine. Natamycin-treated cases were less likely to develop a perforation or need TPK. We also found evidence that natamycin was associated with faster reepithelialization and a slightly smaller scar or infiltrate size from day 7 onwards. There was no evidence of a difference between arms in clearing culture positivity at day 7.

Although chlorhexidine was less effective than natamycin, it is important to view these results within the broader global context, considering frequently limited availability of options and the evidence for their use. The aim of initial FK management is preservation of the eye. Previous studies have reported perforation and TPK rates of 11.1% to 43.8%.^1^^,^^4^^,^^7^^,^^12^^,^^24^ In our study, both treatment arms fared generally better: 5.8% and 13.7% in the natamycin and chlorhexidine arms, respectively. In MUTT1, perforation and TPK rates for natamycin and voriconazole were 11.1% and 21.1%, respectively.^7^

If the eye has been saved, the aim is to achieve the best possible visual acuity. In MUTT1, the mean visual acuity at day 90 was 0.39 logMAR and 0.57 logMAR in the natamycin and voriconazole groups, respectively. This was an improvement of 0.27 logMAR and 0.07 logMAR from baseline for the natamycin and voriconazole groups, respectively.^7^ This compares to 90-day BSCVA of 0.26 logMAR and 0.64 logMAR in the natamycin and chlorhexidine arms, respectively, in our trial, translating to an improvement of 0.29 logMAR in the natamycin arm from baseline and a worsening of 0.03 logMAR in the chlorhexidine arm. Although we cannot make direct comparisons between the 2 trials, these results suggest that chlorhexidine may have comparable effectiveness to topical voriconazole. Unlike MUTT1, which found natamycin more effective than voriconazole for *Fusarium* *spp*. cases,^7^ the difference in BSCVA at day 90 did not vary with causative fungal organism in our trial. It is noteworthy that in clinical practice in many countries, voriconazole is often still used as a first-line or adjunctive agent.^24^^,^^25^

We found that, for patients presenting with mild disease (i.e., good baseline vision and small infiltrate size), there were no cases of poor visual outcome (BSCVA worse than 1.0 logMAR) in either treatment arm, suggesting that chlorhexidine may be an effective treatment for patients presenting early in the course of their disease. Alternatively, it is possible that in some cases, mild fungal infection may be self-limiting and improve regardless of the treatment given. This study was not explicitly designed to test this, and further work is warranted to investigate this further.

There are some potentially relevant differences in eligibility criteria in our study compared with MUTT1. First, we excluded people who were already using antifungals; in MUTT1, 46% of participants had used a topical antifungal before recruitment.^7^ We excluded 88 people from our trial because of prior topical antifungal use, the majority of whom were using natamycin and had been referred to the tertiary center because of deterioration while on this treatment. It is unknown how these would have fared had they been included. Second, MUTT1 recruited people with a visual acuity of between 0.3 logMAR (6/12 Snellen) and 1.3 logMAR (6/120 Snellen).^26^ Our study only excluded people who had no light perception in the affected eye.

Despite natamycin being on the World Health Organization Essential Medicines List, it is still largely unavailable in most of sub-Saharan Africa.^4^ Although our study clearly demonstrates the superiority of natamycin 5% for treatment of filamentous FK, it is important to recognize that in many settings, natamycin and other antifungal eye drops are unavailable. Although chlorhexidine should not be used first-line when natamycin is available, based on the results of this study, there may be situations where cautious use of chlorhexidine might be considered if alternative antifungal treatment is unavailable because without any treatment the eye will likely be lost.^27^

Our results contrast with the 2 earlier trials comparing chlorhexidine with natamycin.^18^^,^^19^ Although these studies had limitations, including small sample sizes, being unmasked, and 1 using half-strength natamycin, they suggested that chlorhexidine could be superior to natamycin in terms of a favorable clinical response at day 5 or “curing” at day 21. Neither study looked at longer-term visual acuity outcomes. Our study, with adequate power, longer follow-up, and a primary outcome of visual acuity, addresses the clinical equipoise raised by these earlier studies and subsequent meta-analysis.^11^

The difference in vision at day 90 between arms likely results from slightly larger scars, more cases of corneal edema, and more persistent EDs in the chlorhexidine group. There was no difference in new cataract development. Prolonged treatment with higher concentrations of chlorhexidine sometimes can be toxic to the corneal epithelium and keratocytes, which may have contributed to these findings, alongside the primary infectious process.^28^ The intensive treatment regimen that we followed for a microbiological cure, although the standard of care for FK, may be more intense than needed. The choice of chlorhexidine 0.2% w/v was based on an earlier pilot trial, which suggested greater efficacy than chlorhexidine 0.02% w/v.^18^ This lower concentration is typically used to treat *Acanthamoeba* keratitis.^29^ It is noteworthy that there were significantly more cases of persistent ED, corneal edema, and delayed reepithelialization in the chlorhexidine arm than the natamycin arm, which could be a result of corneal toxicity resulting from overtreatment with chlorhexidine (either too intensively or with too concentrated a formulation). This may have resulted in more visually significant corneal scarring in the chlorhexidine arm, contributing to the difference in 3-month BSCVA identified by this trial. Further research is necessary to evaluate if lower concentrations of chlorhexidine, or less frequent dosing, are sufficiently effective for treating FK, with the potential advantage of reduced corneal toxicity. It should also be noted that natamycin 5% contains benzalkonium chloride preservatives, whereas chlorhexidine 0.2% is preservative-free; benzalkonium chloride is known to be a broad-spectrum antimicrobial agent and therefore may increase the efficacy of topical natamycin compared with chlorhexidine.^30^

### Study Limitations

Our study has several limitations. It was not possible to mask participants to treatment allocation; however, they were not told which medication they had received, and study-team members were masked to allocation. The primary outcome measure was assessed by an optometrist not otherwise involved in the study. Participants were enrolled in the lowland plains of Nepal, where the pattern of fungal isolates was dominated by dematiaceous fungi; this differs from studies elsewhere, where *Fusarium* and *Aspergillus* are more frequent.^7^^,^^12^^,^^18^^,^^19^^,^^24^ The coronavirus disease 2019 pandemic caused significant disruption, with a 3-month period without participant contact because of restrictions. We reviewed participants as soon as possible after their scheduled follow-up date and included these patients in the primary analysis. This in part may account for the moderate loss to follow-up rate in this study (50/354, 14.1%), although this was less than what was accounted for in the sample size calculation. For adverse effects and clinical outcomes, we have presented multiple *P* values, and it is important to consider that when interpreting these results, one should remember that it is possible to get these results by chance alone; however, given the small size of many of these *P* values and the clear correlation with the conclusive results of the primary outcome, it is unlikely that any correction would alter the results significantly. The 2 treatment arms were generally well balanced in terms of baseline characteristics, although there was some evidence that the severity of disease was worse in the chlorhexidine arm: There were proportionally more patients in this arm who had a history of preceding topical steroid use, the baseline visual acuity was approximately 1 line worse, and there were more culture-positive cases than the natamycin arm; however, our primary analysis adjusted for baseline visual acuity and a sensitivity analysis excluding patients who had used topical steroids showed similar results to the primary analysis. Finally, this trial did not assess the potential role of chlorhexidine as an adjunctive agent to natamycin; this is currently being assessed by our group in East Africa.^31^

In conclusion, natamycin is superior to chlorhexidine for filamentous FK and remains the preferred first-line treatment. This study highlights the need to ensure that it is readily available in all countries where FK is a public health concern. Unfortunately, this is currently far from being the case. Further work is warranted to definitively answer whether lower concentrations of chlorhexidine or combination therapy with natamycin have a role to play in treating FK.
