# Supplementary material for: Topical Chlorhexidine 0.2% versus Topical Natamycin 5% for the Treatment of Fungal Keratitis in Nepal: A Randomized Controlled Noninferiority Trial
Source: Ophthalmology. 2022 May;129(5):530–41. doi: 10.1016/j.ophtha.2021.12.004 (PMC9037000; doi:10.1016/j.ophtha.2021.12.004)
Supplement: Table S1 [file mmc1.pdf]

Table 1: Inclusion and exclusion criteria

Inclusion Criteria (all must be met):	Exclusion Criteria (any of the following):
1. Acute MK characterised by:<br>• Corneal epithelial ulceration >1mm diameter<br>• Corneal stromal infiltrate<br>• Acute inflammation: e.g. conjunctival injection, anterior chamber inflammatory cells, hypopyon.	1. Unwilling/unable to participate in trial and/or attend follow-up
2. Fungal hyphae visualised on smear microscopy and/or *in vivo* confocal microscopy.	2. Aged less than 18 years
3. Agree to be randomised to either treatment arm and are able to give informed consent	3. Pregnancy: self-reported, or by urine human chorionic gonadotropin pregnancy test if uncertain.
4. Agree to be followed up at 2 days, 1 week, 2 weeks, 3 weeks, 2 months, and 3 months	4. Breast feeding self-reported
5. Adults (18 years and older)	5. Prior topical antifungal treatment
	6. No light perception in the affected eye
	7. Fellow eye visual acuity <6/60
	8. Acanthamoebic infection visualised by smear microscopy or IVCM
	9. Clinical evidence of herpetic keratitis
	10. Known allergy to study medication (including preservatives)
	11. Previous keratoplasty in the affected eye
	12. Bilateral corneal ulcers
	13. Very severe ulcers warranting immediate evisceration or conjunctival flap
	14. Endophthalmitis
