# Supplementary material for: Topical Chlorhexidine 0.2% versus Topical Natamycin 5% for the Treatment of Fungal Keratitis in Nepal: A Randomized Controlled Noninferiority Trial
Source: Ophthalmology. 2022 May;129(5):530–41. doi: 10.1016/j.ophtha.2021.12.004 (PMC9037000; doi:10.1016/j.ophtha.2021.12.004)
Supplement: Table S2 [file mmc2.pdf]

Table S2: Numbers of cases confirmed as fungal keratitis by in vivo confocal and/or direct microscopic examination, and causative fungal isolate (to genus level) by treatment arm.

	chlorhexidine (n = 178)		natamycin (n = 176)		Total (N = 354)	
**Microscopy results**						
**IVCM only**	11	(6.2%)	19	(10·8%)	30	(8.5%)
**Microscopy only**	14	(7.9%)	13	(7.4%)	27	(7.6%)
**IVCM and Microscopy**	152	(85.4%)	144	(81.8%)	296	(83.6%)
**Culture Results**						
**No growth (microscopy negative)**	10	(5.6%)	16	(9.1%)	26	(7.3%)
**No growth (microscopy positive)**	23	(12.9%)	24	(13.6%)	47	(13.3%)
**Unidentified filamentous fungi**	18	(10·1%)	16	(9.1%)	34	(9.6%)
***Fusarium* spp.**	25	(14.0%)	22	(12.5%)	47	(13.3%)
***Aspergillus* spp.**	16	(9.0%)	16	(9.1%)	32	(9.0%)
***Curvularia* spp.**	62	(34.8%)	56	(31.8%)	118	(33.3%)
***Bipolaris* spp.**	10	(5.6%)	6	(3.4%)	16	(4.5%)
***Exserohilum* spp.**	2	(1.1%)	5	(2.8%)	7	(2.0%)
***Alternaria* spp.**	3	(1.7%)	2	(1.1%)	5	(1.4%)
***Acremonium* spp./*Sarocladium* spp.**	6	(3.4%)	2	(1.1%)	8	(2.3%)
***Scedosporium* sp.**	0		1	(0·6%)	1	(0·3%)
***Colletotrichum* spp.**	2	(1.1%)	3	(1.7%)	5	(1.4%)
***Purpureocillium* spp.**	0		2	(1.1%)	2	(0·6%)
***Trichoderma* spp.**	1	(0·6%)	2	(1.1%)	3	(0·9%)
**More than one genera**	0		2	(1.1%)	2	(0·6%)
**No culture performed**	0		1	(0·6%)	1	(0·3%)
**Total culture positive**	145	(81·5%)	135	(76·7%)	280	(79·1%)

Data are n (%).
