# Supplementary material for: Topical Chlorhexidine 0.2% versus Topical Natamycin 5% for the Treatment of Fungal Keratitis in Nepal: A Randomized Controlled Noninferiority Trial
Source: Ophthalmology. 2022 May;129(5):530–41. doi: 10.1016/j.ophtha.2021.12.004 (PMC9037000; doi:10.1016/j.ophtha.2021.12.004)
Supplement: Table S3 [file mmc3.pdf]

Table S3. Baseline characteristics and loss-to-follow up (LTFU)

	Lost-to-follow up		Followed up		Total		p-value^
**Age, years**	44.6	(14.6)	47.0	(13.1)	46.7	(13.3)	0·252
**Sex**							
**Male**	19	(39.6%)	116	(37.9%)	135	(38.1%)	0·873
**Female**	29	(60·4%)	190	(62.1%)	219	(61.9%)	
**Infiltrate size, mm**	3.1	(1.6)	2.9	(1.8)	3.0	(1.7)	0·664
**BSCVA, logMAR**	0·53	(0·56)	0·62	(0·61)	0·61	(0·60)	0·316
**Treatment allocation**							
**Chlorhexidine**	25	(52.1%)	153	(50·0%)	178	(50·3%)	0·877
**Natamycin**	23	(47.9%)	153	(50·0%)	176	(49.7%)	

Data are n (%) or mean (SD).

^ P value calculated by Fisher's exact test for categorical variables and t-test for non-categorical variables.
