# Supplementary material for: Topical Chlorhexidine 0.2% versus Topical Natamycin 5% for the Treatment of Fungal Keratitis in Nepal: A Randomized Controlled Noninferiority Trial
Source: Ophthalmology. 2022 May;129(5):530–41. doi: 10.1016/j.ophtha.2021.12.004 (PMC9037000; doi:10.1016/j.ophtha.2021.12.004)
Supplement: Table S4 [file mmc4.pdf]

Table S4: Discordant fungal organisms cultured at baseline and at day 7

Baseline culture result	Day 7 culture result	Comments
Negative	Unidentified filamentous fungus	
Curvularia spp.	*Aspergillus* spp.	
Curvularia spp.	*Aspergillus* spp.	
Curvularia spp.	*Aspergillus* spp.	
Alternaria spp.	*Aspergillus* spp.	Possible contaminant at day 7
Exserohilum spp.	Unidentified filamentous fungus	
Aspergillus spp.	Unidentified filamentous fungus	
