# Supplementary material for: Topical Chlorhexidine 0.2% versus Topical Natamycin 5% for the Treatment of Fungal Keratitis in Nepal: A Randomized Controlled Noninferiority Trial
Source: Ophthalmology. 2022 May;129(5):530–41. doi: 10.1016/j.ophtha.2021.12.004 (PMC9037000; doi:10.1016/j.ophtha.2021.12.004)
Supplement: Table S5 [file mmc5.pdf]

Table S5: Poor outcome (defined as > 1.0 logMAR at day 90 follow up) by treatment arm grouped by quartiles of baseline visual acuity and baseline infiltrate size (mixed infections excluded)

	Top quartile for baseline visual acuity[1]				Second quartile for baseline visual acuity				Third quartile for baseline visual acuity				Bottom quartile for baseline visual acuity			
	Chlorhexidine		Natamycin		Chlorhexidine		Natamycin		Chlorhexidine		Natamycin		Chlorhexidine		Natamycin	
	n	(%)	n	(%)	n	(%)	n	(%)	n	(%)	n	(%)	n	(%)	n	(%)
**Good outcome**	34	(100)	39	(100)	30	(93.8)	39	(97.5)	24	(70·6)	32	(94.1)	12	(29.3)	22	(68.8)
**Poor outcome**	0	(0)	0	(0)	2	(6.2)	1	(2.5)	10	(29.4)	2	(5.9)	29	(70·7)	10	(31.2)

	Top quartile for baseline infiltrate size[1]				Second quartile for baseline infiltrate size				Third quartile for baseline infiltrate size				Bottom quartile for baseline infiltrate size			
	Chlorhexidine		Natamycin		Chlorhexidine		Natamycin		Chlorhexidine		Natamycin		Chlorhexidine		Natamycin	
	n	(%)	n	(%)	n	(%)	n	(%)	n	(%)	n	(%)	n	(%)	n	(%)
**Good outcome**	33	(91.7)	39	(97.5)	28	(93.3)	37	(100)	31	(72.1)	31	(93.9)	8	(25)	25	(71.4)
**Poor outcome**	3	(8.3)	1	(2.5)	2	(6.7)	0	(0)	12	(27.9)	2	(6.1)	24	(75)	10	(28.6)

[1]Top quartile indicates the 25% of patients with the best visual acuity at baseline, or smallest infiltrate size at baseline, as appropriate.
