# Supplementary material for: Topical Chlorhexidine 0.2% versus Topical Natamycin 5% for the Treatment of Fungal Keratitis in Nepal: A Randomized Controlled Noninferiority Trial
Source: Ophthalmology. 2022 May;129(5):530–41. doi: 10.1016/j.ophtha.2021.12.004 (PMC9037000; doi:10.1016/j.ophtha.2021.12.004)
Supplement: Fig S4 [file mmc6.pdf]

Figure 5: Box-plot of mean epithelial defect (ED) size relative to baseline ED size for patients randomised to chlorhexidine (CHX) and natamycin (NATA) at different follow-up intervals

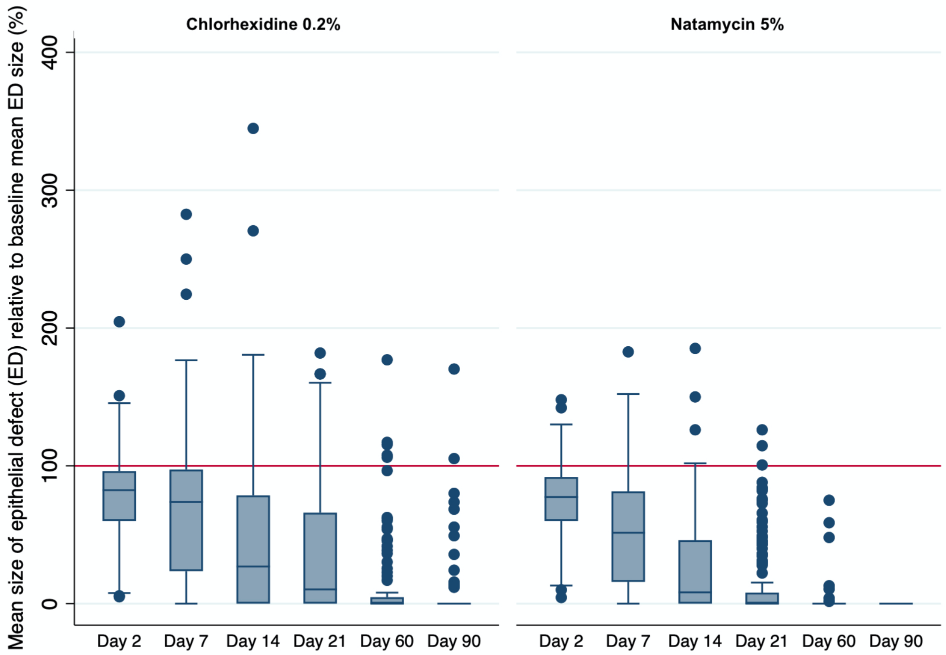

CHX, chlorhexidine 0·2%; NATA, natamycin 5%. Red line represents baseline epithelial defect size. Note patients who had undergone a TPK and/or those who were eviscerated are excluded
